# Distinct Biotypes of Visual Perception in Major Depressive Disorder

**DOI:** 10.1002/advs.74882

**Published:** 2026-03-18

**Authors:** Zhuoran Cai, Xi‐Wen Hu, Yuyang Li, Zheng Lin, Yongjie Li, Yong‐Chun Cai, Tao Xu, Ke Jia, Jin‐Fang Han, Zhong‐Lin Tan, Dezhong Yao, Xue Mei Song, Sugai Liang, Georg Northoff

**Affiliations:** ^1^ School of Medicine Affiliated Mental Health Center & Hangzhou Seventh People's Hospital Zhejiang University Hangzhou China; ^2^ College of Biomedical Engineering and Instrument Science Key Laboratory of Biomedical Engineering of Ministry of Education Qiushi Academy for Advanced Studies Zhejiang University Hangzhou China; ^3^ School of Medicine Department of Psychiatry of the Second Affiliated Hospital Zhejiang University Hangzhou China; ^4^ MOE Key Laboratory For Neuroinformation The Clinical Hospital of Chengdu Brain Science Institute University of Electronic Science and Technology of China Chengdu China; ^5^ Department of Psychology and Behavioral Sciences Zhejiang University Hangzhou China; ^6^ State Key Laboratory of Eye Health Eye Hospital Wenzhou Medical University Wenzhou China; ^7^ University of Ottawa Institute of Mental Health Research University of Ottawa Ottawa Ontario Canada

**Keywords:** biotype, major depressive disorder, machine learning clustering, occipital cortex, visual motion perception

## Abstract

Previous studies showed abnormalities in both visual motion perception (VMP) and occipital cortex activity in subjects suffering from major depressive disorder (MDD). Can the psychophysical and/or neural markers of visual perception serve for clinical identification of MDD subgroups? To address this yet unresolved issue, we develope a novel analytical framework combining visual perceptual measurement with machine learning clustering to identify MDD subgroups. Within a cohort of 272 individuals with acute MDD, this approach reveals a VMP‐positive (VMP‐P) biotype characterized by impaired VMP. Clinically, this biotype exhibits significant cognitive deficits. We validate the robustness of this biotype through cross‐validation and confirm its generalizability in an independent sample (*n* = 63). Furthermore, 7T MRI implicate aberrant neural activity in the occipital cortex as a mechanism underlying the VMP‐P biotype. Our findings establish a novel, clinically translatable path for stratifying MDD, which can guide treatment development and advance precision medicine.

## Graphical abstract

In a discover dataset (272 acute MDD patients), this work identifies a novel depression biotype characterized by impaired visual motion perception, using machine learning clustering. An independent dataset confirms the robustness of this biotype through cross‐validation and demonstrates its generalizability. 7T MRI study further reveals the underlying neural mechanisms, highlighting a specific alteration in occipital cortex function within this biotype.

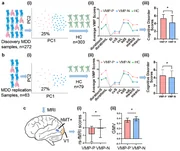

## 1 Introduction

Major depressive disorder (MDD) is a highly prevalent and the leading cause of disability worldwide [1, 2]. Emerging evidence suggests that MDD may consist of distinct subgroups [3, 4], yet few studies have identified reproducible objective markers underlying these subgroups. Notably, cognitive impairment is a core feature of depression, contributing significantly to chronic disability and poor functional outcomes across the lifespan [5, 6, 7, 8] These deficits often persist even after mood symptoms improve in early‐to‐mid adulthood [9] and are associated with reduced antidepressant efficacy in later‐life depression [10, 11]. Given that cognitive dysfunction is a key predictor of treatment resistance [12, 13], impaired social and occupational functioning, and increased suicide risk [14], this study aims to identify a distinct biotype associated with cognitive impairment in depression.

One approach to addressing heterogeneity in depression is to classify it into distinct subgroups [3]. By leveraging machine learning techniques, depression can be prospectively stratified into biotypes—subgroups sharing similar neurobiological dysfunctions [15]—each potentially requiring different treatment strategies [4, 16]. Using this approach, recent studies have identified a cognitive impairment biotype in 27% of depression cases, characterized by dysfunction in the brain‘s cognitive circuits, particularly the dorsolateral prefrontal cortex (dLPFC) [17, 18]. Although MRI‐based neural biomarkers have proven valuable in defining these biotypes [4, 15, 16, 19, 20], their clinical utility remains limited due to high costs, specialized equipment requirements, and the need for expert interpretation [21, 22]. As a more accessible alternative, objective behavioral measurements (e.g., psychophysical experiments) offer a cost‐effective and scalable data modality, facilitating the translation of research into clinical practice [17, 23]. We consequently pursued a two‐step approach. First, we established subtypes of MDD on the ground of objective behavioral‐psychophysical measurements which, second, was further supported neuronal measurements in fMRI.

Impairments in visual motion perception (VMP) may serve as potential markers for multiple neuropsychiatric disorders, including MDD [23, 24, 25, 26, 27, 28, 29]. A well‐established paradigm for assessing VMP function is motion center‐surround interactions, which rely on the visual processing in the human middle temporal complex (hMT+) [27, 30, 31]. This region primarily receives rapid visual input from the primary visual cortex (V1) [32, 33, 34]. Together, both hMT+ and V1 are well known for their roles in visual motion processing [31, 35]. Previous studies have demonstrated significant correlations between VMP indices (e.g., duration thresholds of stimulus grating, suppression index [SI]) and complex cognition scales, such as the performances of working memory and perceptual reasoning [36, 37]. Our recent work further revealed abnormal functional connectivity between visual cortex (VC) and PFC in MDD which underlies the significant relationship between visual perception and cognitive dysfunction [28, 38].

**FIGURE 1:**
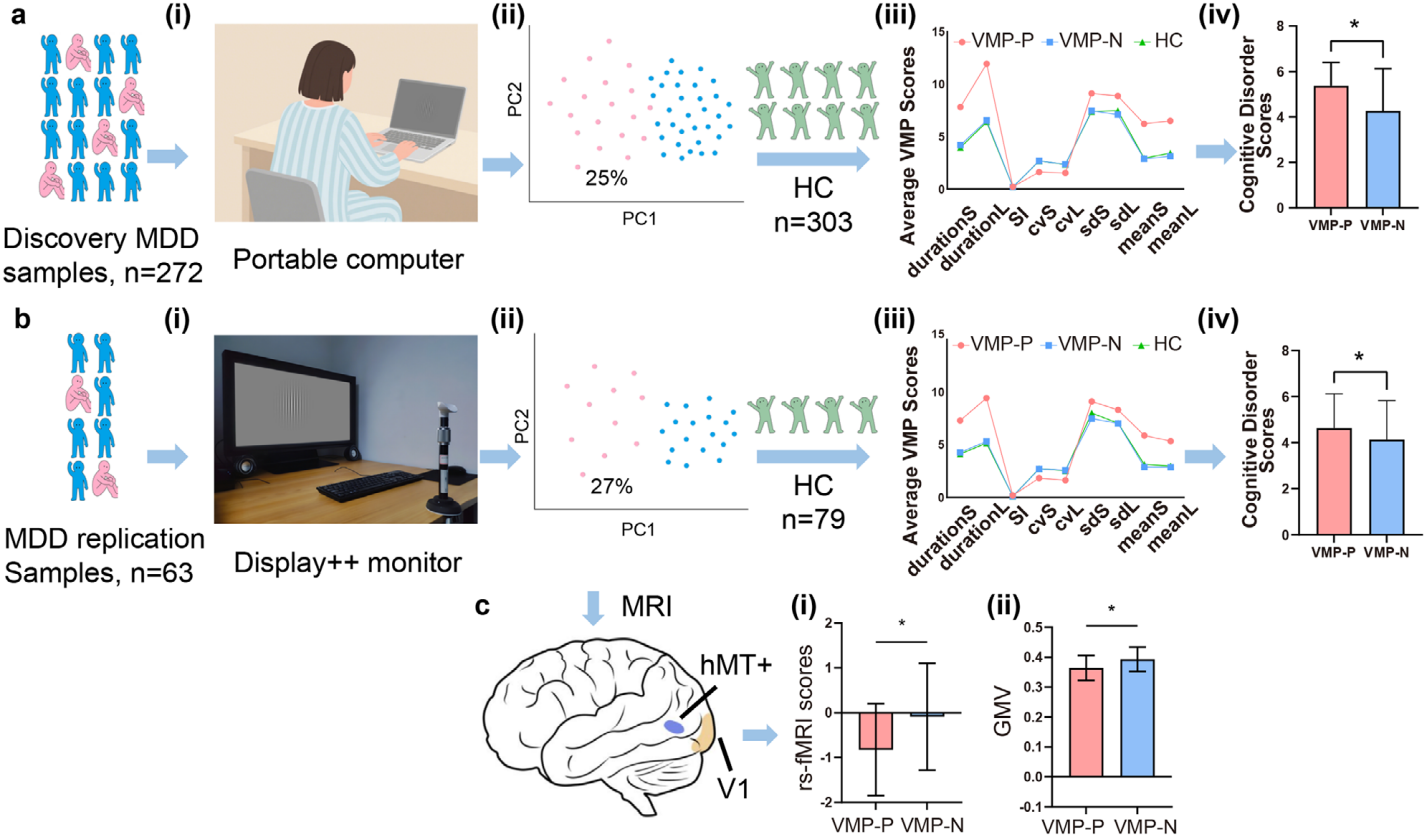
Experimental design. a) The discovery MDD samples (*n* = 272) served to establish an analytical framework for identifying patient subgroups. b) The independent MDD replication samples (*n* = 63) validated the robustness of the stratification. (i) in a and b, Devices for assessing visual perception function. (ii) in a and b, Principal component (PC) clustering was used to delineate two major depressive disorder (MDD) subgroups, designated as MDD1 (pink dots) and MDD2 (blue dots). (iii) in a and b, Compared the nine‐vision motion perception (VMP) indices across the MDD1, MDD2, and HC groups. These indices included three standard static measures: the duration threshold for a small stimulus (durationS), for a large stimulus (durationL), and the suppression index (SI). The remaining six were variability indices, calculated for each stimulus size: the mean (meanS, meanL), standard deviation (sdS, sdL), and coefficient of variation (cvS, cvL). Significant differences from HC group were observed on nearly all VMP indices (except for SI values) in the MDD1 group (pink line). We therefore classified this group as the VMP‐positive (VMP‐P) biotype, characterized by marked VMP impairment. Conversely, the MDD2 group (blue line) demonstrated performance comparable to the HC group and was classified as the VMP‐negative (VMP‐N) biotype. (iv) in a and b, Compared the clinical symptom between the VMP‐P and VMP‐N biotypes. c) The 7T MRI study of the replication sample revealed the neural mechanistic underpinning in the occipital cortex, including hMT+(shows with blue color) and V1(shows with yellow color) brain regions. (i) and (ii) in c, Respectively represents the comparison between the two biotypes (VMP‐P and VMP‐N) in rs‐fMRI and grey matter volume (GMV) indicators.

In this study, we employed a k‐means clustering algorithm [17, 23, 39, 40] and VMP measurements [23, 24, 25, 26, 27, 28, 29, 30, 31] to identify a VMP‐positive (VMP‐P) biotype—characterized by impaired VMP function—in a large acute MDD samples (discovery dataset, *n* = 272) (Figure 1a). We hypothesized that impaired VMP function could serve as a prospective biomarker for cognitive impairment tendency in these patients. To validate our findings, we replicated the clustering approach in an independent acute MDD samples (replication dataset, *n* = 63, Figure 1b) collected across different sites and with different devices (see Methods). Additionally, the smaller replication cohort underwent 7T MRI to investigate neural mechanisms associated with the occipital cortex (including hMT+ and V1 brain regions) (Figure 1c). Given the critical roles of hMT+ and V1 in VMP function, we further hypothesized that the VMP‐P biotype would exhibit neural abnormalities in these two brain regions.

Our findings demonstrate that depressed patients with significant cognitive dysfunction can be identified based on measurements of VMP function and machine learning clustering. The robustness of these results was confirmed through cross‐validation, and their generalizability was further supported using an independent replication dataset. These findings align with our hypothesis that simple perceptual dysfunction (e.g., VMP impairment) can predict complex cognitive deficits in MDD patients. Additionally, MRI analyses revealed the neural mechanisms underlying these results and a mediation model further demonstrated that abnormal resting‐state fMRI (rs‐fMRI) indicators in occipital regions act as mediators, linking impaired visual perception to cognitive deficits in the VMP‐P biotype of depression.

Together, our results identify a novel MDD biotype characterized by impaired visual motion perception (VMP‐P), which is associated with significant cognitive dysfunction. The neural basis of this biotype, particularly the role of the occipital cortex, is further supported by our 7T MRI study. These findings pave the way for improved diagnosis and targeted treatments for cognitive dysfunction in MDD, advancing precision medicine in clinical practice.

## 2 Results

Our study proceeded in two stages to identify and validate distinct MDD subgroups based on VMP indices. The primary, larger sample (272 acute MDD and 303 HC; demographic details in Table 1) served as a discovery cohort to establish the clustering model. A second, smaller sample (63 acute MDD and 79 HC; Table S1) was used as a replication cohort to validate the subgroup structure. Although VMP measurements were collected at different sites with different devices (Figure 1a,b), the clustering results proved consistent across both cohorts. The replication cohort also participated in a 7T MRI study, allowing us to explore the neural mechanisms associated with the VMP‐defined subgroups (Figure 1c).

**TABLE 1:** Demographics and patient clinical data in the discovery dataset.

Variables	MDD patients (*n* = 272)	Healthy controls (*n* = 303)	*p* value
Gender(M/F)	69/203	102/201	0.11
Age, years (SD)	25.33 (6.609)	24.88 (4.496)	0.33
HAMD‐17 scores (SD)	21.07 (3.713)	—	—
Cognitive disturbance scores (SD)	4.250(1.665)	—	—
Retardation scores (SD)	6.934(1.426)	—	—
Anxiety scores (SD)	4.680(1.622)	—	—
Sleep disturbance scores (SD)	2.864(1.603)	—	—
Weight loss scores (SD)	0.4154(0.6981)	—	—

### 2.1 Visual Perception Indices Define Two Biotypes

Following previous studies [23, 24, 25, 26, 27, 28, 29, 30, 31], we used a standard motion discrimination task (VMP paradigm) to investigate visual psychophysical performance. Performance was evaluated using both standard (static) [24, 25, 26, 27, 28, 30, 31] and novel dynamic indices [23, 29]. The standard performance index in this paradigm is the duration threshold—the stimulus exposure time required to achieve a predetermined steady‐state accuracy rate, derived by fitting data from multiple trials (see Methods). This yields three standard (static) indices: the duration threshold for a small stimulus (durationS), for a large stimulus (durationL), and a suppression index (SI) (Figure S1a). However, these static indices fail to capture intra‐subject variability—the trial‐to‐trial fluctuations in visuo‐behavioral responses [23, 29]. To quantify this variability, we derived a new time series by calculating the absolute difference in grating duration between all pairs of neighboring trials, excluding the initial maximum and minimum values presented at the start of the experiment (for details, see Methods). From this series, we derived six variability indices for both small and large stimulus sizes: the mean (meanS, meanL), standard deviation (sdS, sdL), and coefficient of variation (cvS, cvL) [23, 29](Figure S1b). Together with the three static measures, these amounted to a total of 9 VMP indices.

To identify data‐driven subgroups based on VMP indices, we performed principal component analysis (PCA) on the 9 VMP indices from the larger MDD patients (*n* = 272). The first two principal components (PC1: 47.87%, PC2: 17.91%; cumulative variance = 65.78%) were retained for subsequent clustering (Figure S2a1,b1). We then used these two PCs as input for a k‐means algorithm and evaluated solutions between 2 and 10 clusters. The consensus from a distinct elbow in the scree plot and a peak in the silhouette metric [16, 17, 41] (Figure S3a1,b1) confirmed k = 2 as optimal. In the replication MDD sample, the first two principal components (PC1: 46.26%, PC2: 19.57%; cumulative variance = 65.83%) were retained for subsequent clustering (Figure S2a2,b2). The optimal number of components was identified by a distinct elbow in the scree plot, which coincided with a peak in the silhouette score (Figure S3a2,b2).

### 2.2 Biotype Validation

**FIGURE 2:**
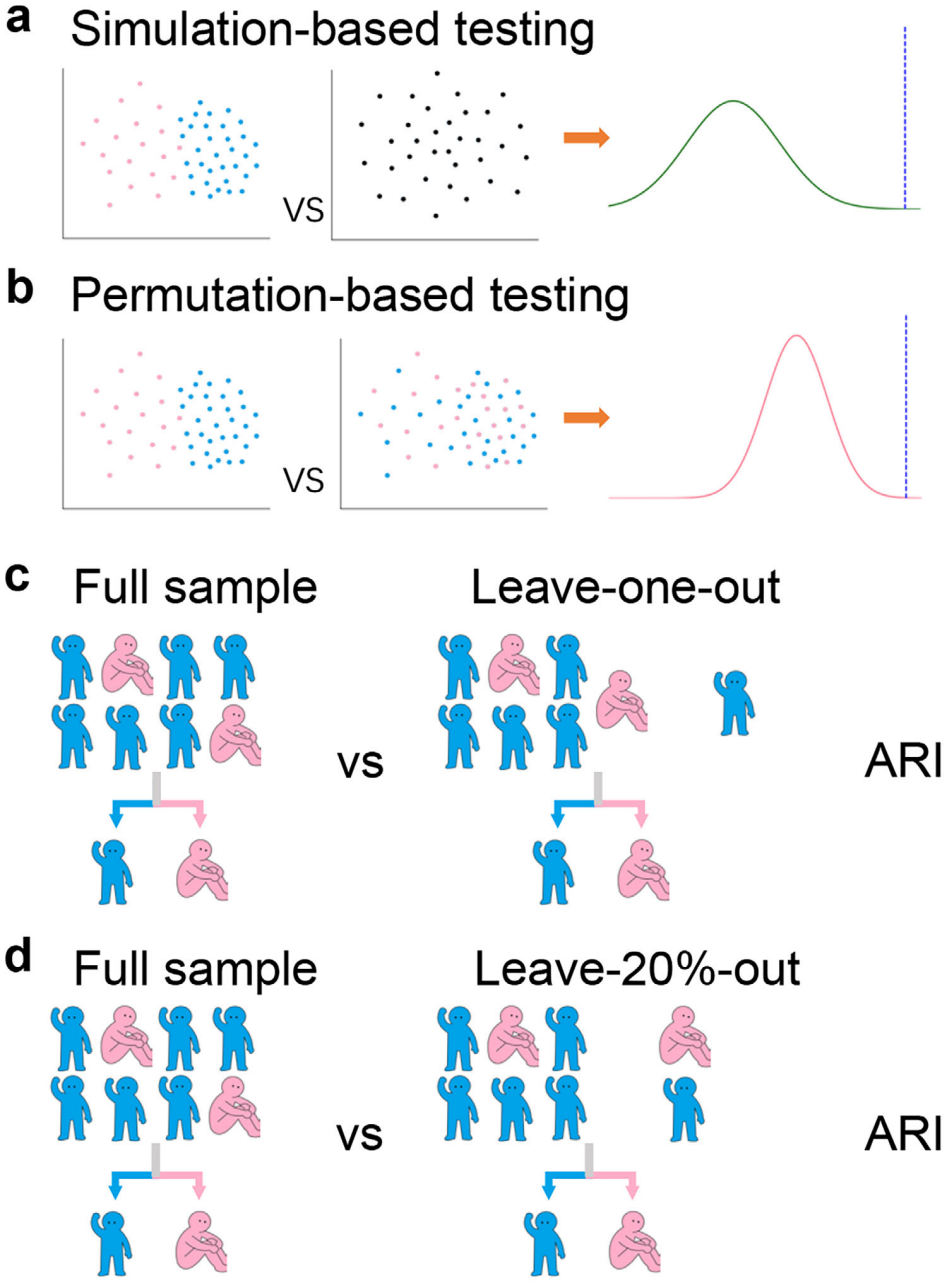
Overview of biotype validation. a) The silhouette index of the identified clustering solutions was evaluated against a null model of multivariate normal distribution with conserved covariance between individuals. b) The silhouette index was compared against values derived from data with randomly permuted participant labels. c and d) Cluster stability was assessed via resampling validation: leave‐one‐out cross‐validation (c) and leave‐20%‐out cross‐validation (d). For each iteration, consistency between the original biotype assignments and those from resampled datasets was quantified using the Adjusted Rand Index (ARI).

To validate the optimal number of clusters and assess the statistical significance of the clustering solution, we employed three distinct testing approaches (Figure 2): a simulation‐based test, a permutation‐based test and cross‐validation [4, 16].

In both the discovery and replication datasets, the silhouette index for clustering solutions with two or three clusters was significantly greater than that from both a multivariate normal null model and a permuted null model (all *P* < 0.05; Figures S4–S6). Cross‐validation demonstrated good stability for all clustering solutions in both the discovery and replication datasets. In the discovery set, the adjusted Rand index (ARI) was >0.75 for leave‐one‐out and >0.70 for leave‐20%‐out validation (Figure S7a). The independent replication set also showed stable results, with ARIs of >0.60 and >0.50 for the respective validation methods (Figure S7b).

In both the discovery and independent replication data sets, a two‐cluster solution was consistently identified as optimal. This solution demonstrated strong validity, with significant silhouette index tests against both multivariate normal and permuted null models (all *P* < 0.01). It also exhibited high stability in cross‐validation analyses, with Adjusted Rand Index (ARI) values exceeding 0.93 in leave‐study‐out and leave‐20%‐out tests. To evaluate the consistency of the clustering results obtained from a discovery dataset and a replication dataset, this study employed a validation strategy based on the correlation analysis of cluster indicator. The reclassification accuracy was 92.06% when using the discovery dataset as the reference standard, and 84.19% when using the replication dataset as the reference (Table S2).

**FIGURE 3:**
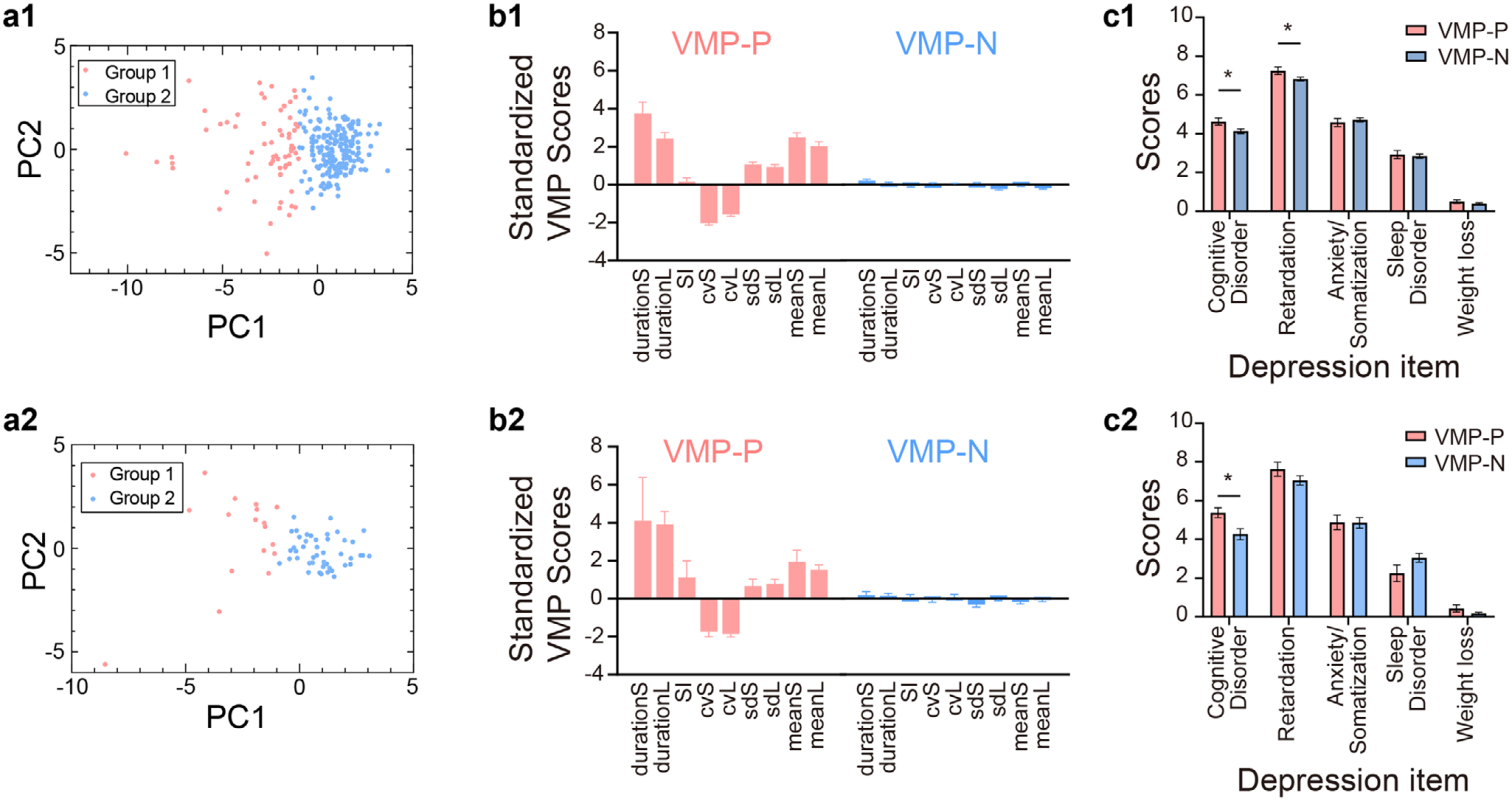
Identifying MDD subgroups, and VMP indices/clinical symptoms scores in the two subgroups. a1 and a2) Following PCA, k‐means clustering identified two subgroups from discover (a1) and replication (a2) MDD datasets. b1and b2) The VMP‐P biotype characterized by VMP impairment, on the contrary, the VMP‐N biotype demonstrated performance within the health range. The y‐axis represents the standardized VMP indices (normalized with the corresponding values of HC groups). The 9 VMP indices are: durationS, durationL, SI, cvS, cvL, sdS, sdL, meanS, meanL, details see the text and methods. c1and c2) The two MDD biotypes with distinct clinical profiles. In the discovery dataset (c1), the VMP‐P biotype exhibited higher scores for cognitive disorder (t = 2.149, *p* = 0.03) and psychomotor retardation (t = 2.129, *p* = 0.03) than the VMP‐N biotype. This finding was partially confirmed in the replication dataset (c2), where the VMP‐P biotype again showed significantly greater cognitive disorder (t = 2.245, *p* = 0.03), but the difference in psychomotor retardation was no longer significant. For both cohorts, symptoms of anxiety, sleep disturbance, and weight loss did not differ significantly between the two biotypes (all *p* > 0.05, c1 and c2).

In the discovery dataset, the 2‐cluster analysis identified two distinct subgroups (Table S3a): a smaller cluster (MDD1, *n* = 67, 24.6%) characterized by marked VMP impairment, termed the VMP‐positive (VMP‐P) biotype, and a larger cluster (MDD2, *n* = 205, 75.4%) with performance within the healthy range, termed the VMP‐negative (VMP‐N) biotype (Figure 3a1). The two distinct biotypes differed significantly on all VMP indices (*P_FDR_* < 0.001) except for the SI value (*P_FDR_* = 0.07) (Figure 3b1). This biotype structure was successfully replicated in an independent cohort, which also yielded a VMP‐P biotype (*n* = 17, 27.0%) and a VMP‐N biotype (*n* = 46, 73.0%) (Figure 3a2,b2; Table S3b). As in the discovery set, the two biotypes in the replication cohort differed significantly on all VMP indices (*P_FDR_* < 0.05) except for the SI value (*P_FDR_* = 0.11) (Figure 3b2). The validity of this classification was further supported by joint principal component analysis (JPCA). When analyzing healthy controls (HC) together with the MDD biotypes, we observed no statistically significant differences between the HC group and the VMP‐N biotype in both the discovery and replication datasets, reinforcing that the VMP‐N biotype's performance is within a healthy range (Figure S8a,b).

We also performed clustering without applying PCA for comparison. As presented in Table S4 and Figure S9, the two approaches—clustering on the original variables and clustering on the top two principal components—yielded highly consistent results, with only a minimal proportion of participants being classified differently. To facilitate clearer clinical interpretation and application, we selected the clustering results based on the top two principal components.

### 2.3 Clinical Symptoms of Biotypes

To assess whether the depression biotypes defined by VMP indices were associated with distinct clinical profiles, we first compared the severity of depressive symptoms across the clusters in the discovery set (*n* = 272), as measured by the HAMD‐17 scale. Symptoms were grouped into five domains (Figure 3c1): cognitive impairment, psychomotor retardation, anxiety/somatization, sleep disturbance, and weight loss. Although the two biotypes (VMP‐P and VMP‐N) did not differ in their total HAMD‐17 scores (t = 1.762, *p* = 0.08; Figure S10a), we observed distinct symptom profiles between them. The VMP‐P biotype exhibited significantly higher scores for cognitive impairment (t = 2.149, *p* = 0.03) and psychomotor retardation (t = 2.129, *p* = 0.03). In contrast, scores for anxiety, sleep disorder, and weight loss were comparable between the two MDD subgroups (all *p* > 0.05; Figure 3c1). In a subset of the discovery cohort (*n* = 48), patients with the VMP‐P biotype (*n* = 10) scored significantly lower on the Block Design Test (BDT)—a measure of perceptual reasoning [36, 37]—than those with the VMP‐N biotype (*n* = 38) (Figure S11). These results further establish cognitive impairment as a defining characteristic of the VMP‐P biotype.

In the replication set, the comparison of HAMD‐17 scores also revealed that the VMP‐P biotype exhibited significantly greater cognitive disorder scores (t = 2.245, *p* = 0.03) than the VMP‐N biotype. In contrast, we found no significant differences in the scores of psychomotor retardations, anxiety, sleep disturbance and weight loss (all *p* > 0.05) (Figure 3c2). Total HAMD‐17 scores also did not differ significantly between the two biotypes (t = 1.009, *p* = 0.32; Figure S10b). This shows remarkable similarity in the results of both discovery and replication data sets as both show that the VMP‐P biotype exhibits more severe cognitive deficits.

### 2.4 Neuroimaging Features in Occipital Cortex of Biotypes

The replication sample (acute MDD: *n* = 63; HC: *n* = 79) underwent 7T MRI to investigate the neural mechanisms of the biotypes, with a focus on the VMP‐P biotype. We hypothesized that the VMP‐P biotype (*n* = 17, within the replication sample) would exhibit neural abnormalities in the occipital cortex (including V1 and hMT+ brain regions).

**FIGURE 4:**
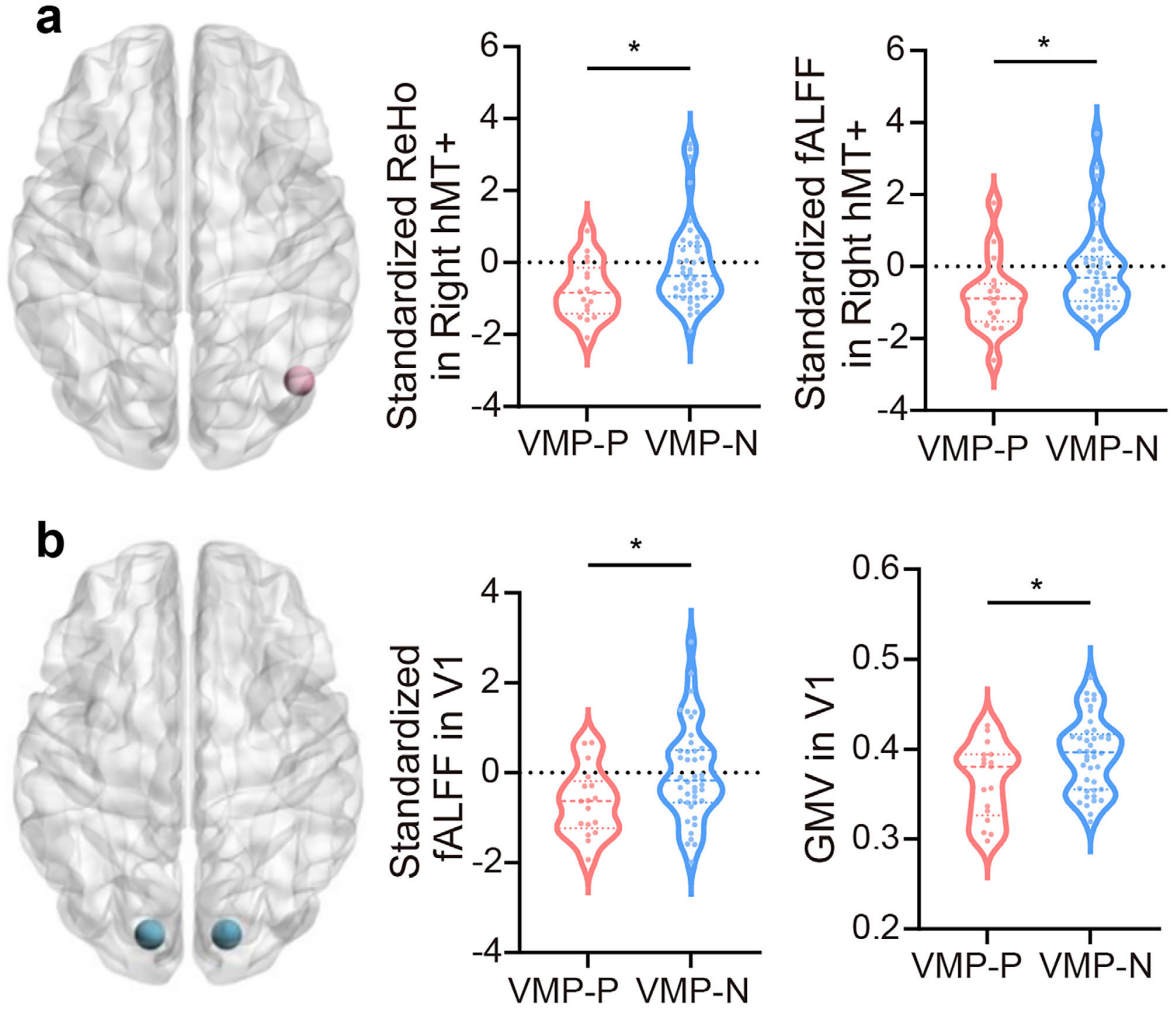
Differences in neuroimaging indicators between the two biotypes in the hMT+ (a) and V1 (b) regions. a) Left column: A diagram of the right hMT+ brain region. Middle column: The VMP‐P biotype showed significantly reduced fALFF in the right hMT+ compared to the VMP‐N biotype (t = 2.237, *p* = 0.03). Right column: The VMP‐P biotype showed significantly reduced ReHo in the right hMT+compared to the VMP‐N biotype (t = 2.200, *p* = 0.03). b) Left column: A diagram of the bilateral V1 brain regions. Middle column: The VMP‐P biotype had significantly lower fALFF in the bilateral V1 compared to the VMP‐N biotype (t = 2.244, *p* = 0.03). Right column: The VMP‐P biotype had significantly reduced GMV in the bilateral V1 compared to the VMP‐N biotype (t = 2.468, *p* = 0.02). In all panels, the dotted line represents the standardized indices of the healthy control (HC) subjects.

We assessed local spontaneous neural amplitude variability and intra‐regional synchronization within the occipital cortex (V1 and hMT+) using fractional amplitude of low‐frequency fluctuations (fALFF) and regional homogeneity (ReHo) [42, 43], standardized to HC indices. In the hMT+ brain regions, the VMP‐P biotype exhibited significantly lower values in both neural activity variability (fALFF) and neural synchronization (ReHo) than the VMP‐N biotype. This effect was lateralized: the right hMT+ showed significantly reduced fALFF (t = 2.237, *p* = 0.03) and ReHo (t = 2.200, *p* = 0.03) in the VMP‐P group (Figure 4a), while left hMT+ values did not differ significantly (fALFF: *p* = 0.19; ReHo: *p* = 0.31; Figure S12a,b). Grey matter volume (GMV) in the left hMT+ did not differ between the two biotypes (t = 0.18, *p* = 0.86). In the right hMT+, however, the VMP‐P biotype showed a trend‐level reduction compared to the VMP‐N biotype (t = 1.80, *p* = 0.08) (Figure S12c,d). In contrast, V1 regions showed a different pattern. The VMP‐P biotype had significantly lower fALFF in bilateral V1 (t = 2.244, *p* = 0.03) and significantly reduced GMV (t = 2.468, *p* = 0.02) compared to the VMP‐N biotype (Figure 4b). The VMP‐P biotype had lower ReHo in bilateral V1 than the VMP‐N biotype, but these differences were not statistically significant (left: *p* = 0.06; right: *p* = 0.19; Figure S12e,f).

Together, these results indicate that the VMP‐P biotype of MDD is characterized by diminished local spontaneous activity dynamics in both hMT+ and V1 brain regions including reduced regional variability and synchronization specifically in the right hMT+, and structural atrophy in V1, compared to the VMP‐N biotype.

### 2.5 Associations Between Clinical Symptoms, Visual Perception, and Neuroimaging in the VMP‐P Biotype

**FIGURE 5:**
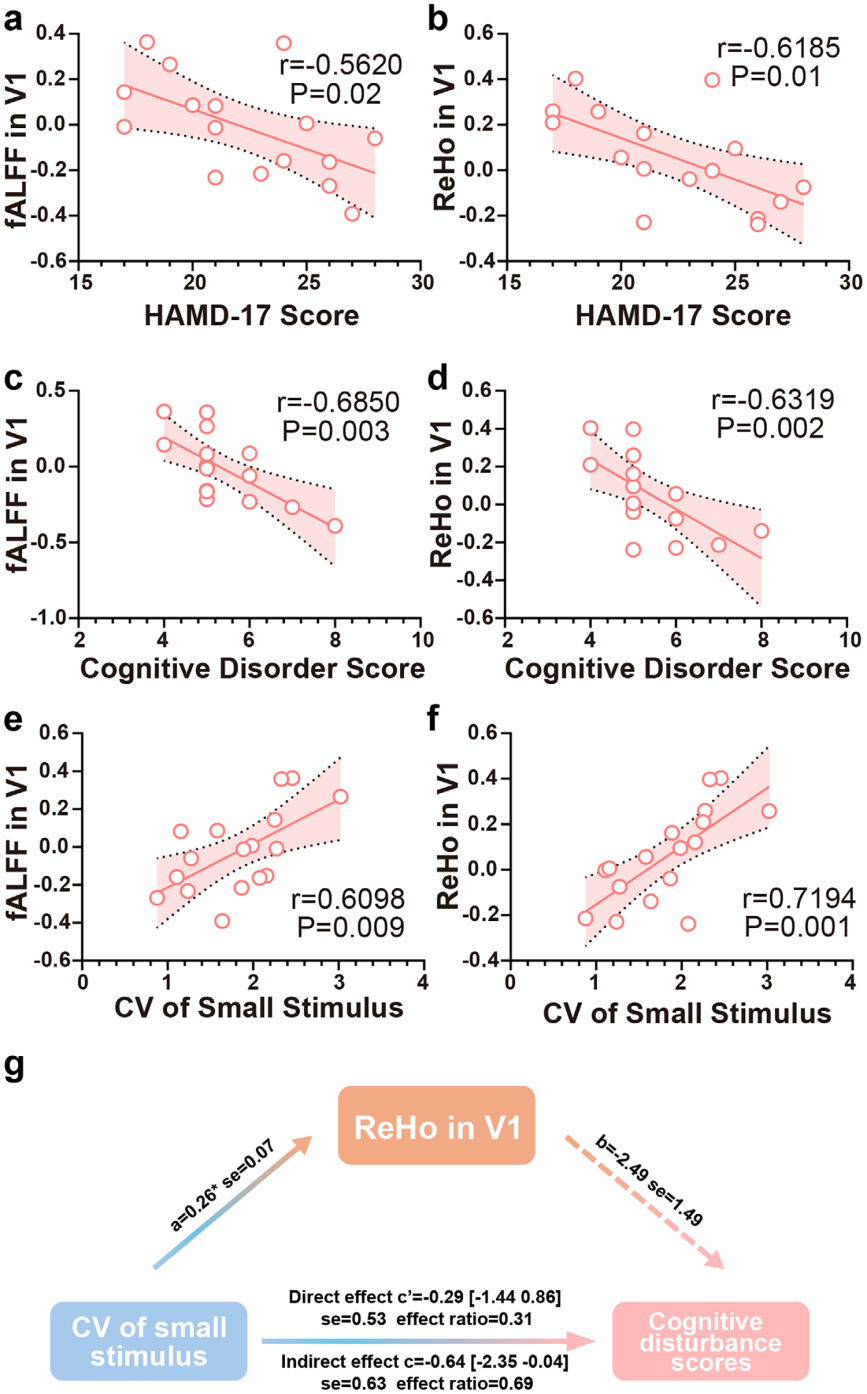
The relationships between rs‐fMRI indicators, VMP indices, and clinical scores within the VMP‐P biotype. a and b) Negative correlations were observed between HAMD‐17 scores and both the regional homogeneity (ReHo) (a) and fractional amplitude of low‐frequency fluctuations (fALFF) (b) in the primary visual cortex (V1). c and d) Cognitive disorder scores were negatively correlated with V1 ReHo (c) and V1 fALFF (d). e and f) The cvS VMP indices showed significant positive correlations with both V1 ReHo (e) and V1 fALFF (f). g) Mediation analysis among the three variables of cvS, V1 ReHo and cognitive disorder scores, which yields that the relationship between cvS and cognitive disorder scores was indirect‐only and mediated by the intra‐regional synchronization (ReHo) in V1.

To investigate relationships between neural mechanisms, clinical symptoms, and VMP performance in the VMP‐P biotype of MDD, we performed Pearson correlation analyses and visualized them in a matrix (Figure S13). We found significant negative correlations between clinical symptoms (total HAMD‐17 scores and cognitive disorder scores) and V1 spontaneous activity dynamics scores. Specifically, total HAMD‐17 scores correlated negatively with activity dynamics in V1 ReHo (*r* = −0.619, *p* = 0.01) and V1 fALFF (*r* = −0.5620, *p* = 0.02) (Figure 5a,b). Similarly, cognitive disorder scores correlated negatively with activity dynamics of V1 ReHo (*r* = −0.632, *p* = 0.002) and V1 fALFF (*r* = −0.685, *p* = 0.003) (Figure 5c,d). Conversely, cvS VMP indices showed significant positive correlations with V1 ReHo (*r* = 0.719, *p* = 0.001) and V1 fALFF (*r* = 0.610, *p* = 0.009) (Figure 5e,f). Given these correlations, we undertook mediation analyses to explore potential pathways [44]. A significant mediation effect was found for one model: V1 ReHo mediated the relationship between cvS values and cognitive disorder scores. The indirect effect of cvS values on cognitive disorder scores through V1 ReHo was significant (c = −0.64, bootstrapped 95% CI [−2.35, −0.04]), while the direct effect was not (c' = −0.29, 95% CI [−1.44, 0.86]), indicating full mediation (Figure 5g). V1 ReHo accounted for approximately 69% of the total effect. For sensitivity, we tested the alternative model with cvS values as the mediator. There was no significant mediation effect (c = −0.59, bootstrapped 95% CI [−2.44, 3.01]) (Figure S14). However, in the VMP‐N biotype, we found no significant correlations between the above V1 spontaneous activity scores, clinical symptoms and VMP performance (Figure S15a–f).

Together, our results reveal that in the VMP‐P biotype, visual perception deficits—linked to occipital cortex abnormalities—were associated with broader cognitive impairment. Mediation analysis indicated that the regional homogeneity (ReHo) of the primary visual cortex (V1) mediates the relationship between impaired visual perception and higher‐order cognitive dysfunction.

## 3 Discussion

In this study, we identified two biotypes of MDD linked to visual perceptual processing—VMP‐P and VMP‐N—using a machine learning clustering algorithm. The VMP‐P biotype, characterized by impaired VMP function, represented about a quarter of our MDD sample, while the VMP‐N biotype demonstrated VMP performance within the healthy range. Clinically, the VMP‐P biotype exhibited significantly greater cognitive disturbance than the VMP‐N biotype. We first identified these biotypes in a large discovery sample and confirmed their robustness through cross‐validation. Their generalizability was further demonstrated in an independent, replication sample. Additionally, a 7T MRI study within the replication cohort revealed distinct neural mechanisms in the occipital cortex underlying the two biotypes. Together, we identified a novel biotype (VMP‐P) associated with cognitive impairment in MDD. These findings position VMP indices as a promising objective marker linking to cognitive dysfunction in MDD, which may guide new precision treatment strategies.

While structural and functional MRI have been highly successful in identifying biomarkers of brain structure, function, and psychopathology [4, 15, 16], their clinical adoption is constrained by high costs, a reliance on specialized equipment, and the need for significant technical expertise [21, 22]. To address these limitations, we present a more accessible alternative: a 10‐minute visual psychophysics (VMP) paradigm that collects objective behavioral data from MDD patients. This method is not only cost‐effective and easy to administer but also provides a valuable complementary data modality. It enabled the identification of a distinct VMP‐P biotype—characterized by impaired VMP function (Figure 3a1,b1)—within a larger MDD cohort (*n* = 272) (Figure 1a). Previous studies, including our own, have reported significant correlations between VMP indices (e.g., durationS, durationL) and performances on cognitive tasks, suggesting a link between visual perceptual processing and higher‐order cognition [36, 37, 45]. Our present results demonstrate that the VMP‐P biotype exhibits significantly higher cognitive disturbance scores than the VMP‐N biotype in the larger samples (Figure 3c1; Figure S11b). These results establish a novel method for clustering depression subgroups. To demonstrate robustness, we successfully replicated our findings in an independent data set (Figures 1b and 3a2–c2) and through cross‐validation (Figure S7).

Furthermore, a 7T MRI study revealed the neural mechanistic underpinning within the occipital cortex including V1 and hMT+ (Figures 4 and 5). We found that the VMP‐P biotype exhibited significantly reduced local spontaneous activity dynamics (fALFF) in the hMT+ and V1 areas compared to the VMP‐N biotype (Figure 4a,b). Furthermore, regional homogeneity (ReHo) was lower in the VMP‐P group, specifically in the right hMT+ (Figure 4a) and the left V1 (a difference that approached significance, *p* = 0.06; Figure S12e). These results align with a previous pilot study [46]. The study reported significantly lower spontaneous neural activity dynamics with reduced regional homogeneity (ReHo) in the calcarine sulcus (as an essential part of the V1 cortex) of depression patients relative to healthy controls. The authors posited that this neural deficit underlies the diminished visual and cognitive processing capabilities [46].

We also found significant GMV atrophy in V1 of the VMP‐P biotype compared to the VMP‐N biotype. Given the established association between V1 GMV and working memory performance [47], this structural difference may underlie the more severe cognitive deficits characteristic of the VMP‐P biotype. Our results demonstrate that GMV patterns differ significantly among MDD biotypes, providing further evidence that anatomical brain atrophy can serve as an objective marker for MDD stratification [4, 15, 48, 49, 50, 51, 52, 53]. Future studies should investigate the utility of GMV as an objective indicator for distinguishing cognitive impairment biotypes in depression.

The mediation model indicated that V1 ReHo mediates the relationship between impaired visual perception and higher‐order cognitive dysfunction in the VMP‐P biotype of MDD (Figure 5g). This suggests that abnormal neural activity in V1 may be an important mechanism underlying these impairments. Consequently, interventions targeting this activity (e.g., normalizing V1 fALFF and ReHo) may potentially reverse both the perceptual and cognitive deficits in MDD.

**FIGURE 6:**
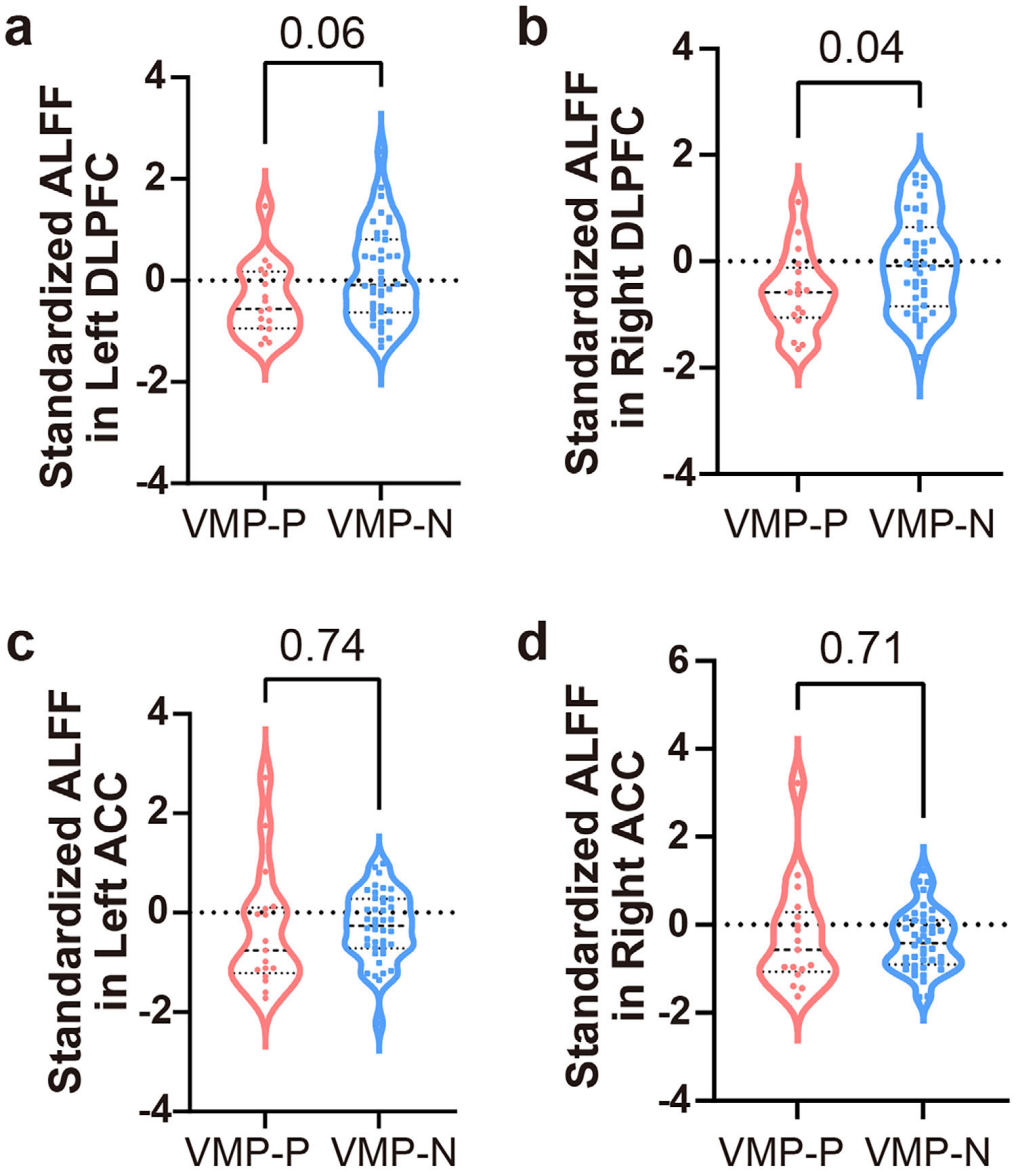
Neuroimaging analyses identified differences ALFF within the DLPFC and ACC between the two biotypes. Compared to the VMP‐N biotype, the VMP‐P biotype demonstrated a trend toward lower ALFF in the left DLPFC (t = 1.939, *p* = 0.06) (a) and significantly lower ALFF in the right DLPFC (t = 2.122, *p* = 0.04) (b). No significant differences were found in the ACC (left: t = 0.333, *p* = 0.74; right: t = 0.382, *p* = 0.70) (c and d). In all panels, the dotted line represents the standardized indices of the healthy control (HC) subjects.

A growing body of research links basic perceptual and complex cognitive processes [37, 54, 55, 56, 57]. In line with this framework, we complementarily examined spontaneous neural activity (amplitude of low‐frequency fluctuations, ALFF) in key cognitive control regions: the dorsolateral prefrontal cortex (DLPFC) and anterior cingulate cortex (ACC) [17, 18]. Compared to the VMP‐N biotype, the VMP‐P biotype showed significantly lower ALFF in the right DLPFC (t = 2.122, *p* = 0.04) and a trend toward lower ALFF in the left DLPFC (t = 1.939, *p* = 0.06) (Figure 6a,b). In contrast, ALFF in bilateral ACC did not differ between biotypes (left: t = 0.3329, *p* = 0.74; right: t = 0.3817, *p* = 0.70) (Figure 6c,d). These findings suggest functional abnormalities in both unimodal visual cortex (VC) regions (periphery) and transmodal DLPFC regions (core) in the VMP‐P biotype, which is in line with the observation that topographic unimodal transmodal changes in MDD studies [58, 59, 60].

While cognitive impairment is a known contributor to poor functional outcomes and reduced efficacy of first‐line antidepressants [5, 17], non‐pharmacological interventions like repetitive TMS (rTMS) have demonstrated promising efficacy in ameliorating these deficits [61, 62, 63, 64]. Emerging evidence suggests that stimulation of the left visual cortex (VC) may be non‐inferior to dorsolateral prefrontal cortex (DLPFC) stimulation in improving both depressive symptoms and cognitive function [65, 66]. Recent studies have reported that rTMS can alter brain function and effectively improve symptoms of depression by increasing spontaneous neural activity and regional homogeneity (ReHo) in several brain regions, including the occipital cortex [67, 68]. Further research on neural stimulation indicates that targeting either the left DLPFC or the left VC can strengthen functional connectivity between these regions, leading to improved cognitive performance in patients with depression [69, 70]. Collectively, these findings imply that TMS applied to the VC region may help restore typical brain topography by modulating, and thus re‐organizing the pathological shift from unimodal (e.g., VC) to transmodal (e.g., DLPFC) regions observed in MDD patients [71].

TMS offers a key advantage over pharmacologic methods by enabling the direct targeting of dysfunctional brain circuits, positioning it as a leading approach for precision psychiatry [72]. A growing pool of studies have demonstrated that identifying rTMS targets based on neuroimaging biomarkers can lead to personalized stimulation sites, which are associated with improved patient response [18, 66, 73, 74, 75, 76]. Notably, a recent study used task‐fMRI biomarkers to stratify cognitive impairment into biotypes, demonstrating that this classification can predict TMS‐related cognitive improvement [18]. Our results in this study identify a novel biotype of MDD (VMP‐P), defined by impaired visual perceptual function and associated with cognitive impairment (Figure 3), which presents a promising target for rTMS treatment. Based on the neural abnormalities observed in visual cortex (VC, including hMT+ and V1 regions) of the VMP‐P biotype (Figure 4a,b), we propose that rTMS over VC could be a potential treatment option for visual perceptual/cognitive impairment in the biotype of MDD patients. Since therapeutic rTMS is typically used to once pharmacotherapy fails [17, 18], the VMP‐P biotype identified in this study could help clinicians select patients who are most likely to benefit from TMS treatment. This would optimize improvements in both visual perception/cognition and depressive symptoms.

Some limitations need to be mentioned. First, we calculated the cognitive disorder scores with different item numbers based on the HAMD scale (see Methods), other scales dedicated specifically to cognitive impairment like the MATRICS Cognitive Consensus Battery (MCCB) and the Cambridge Neuropsychological Automated Testing Battery (CANTAB) [77, 78, 79] may want to be used in the future. Second, as nearly all patients with acute MDD in our datasets were medicated (Table S5), we investigated the potential influence of psychotropic drugs. Following established standards [80, 81], we calculated a medication load score (0 for no medication, and 1–3 for dose‐equivalents below, equal to, or above the mean effective daily dose). Overall, we found minimal association between medication status and our VMP/imaging biomarkers [27, 28, 29]. Correlation analyses revealed no significant relationships between biomarker variables and the load codes for either antidepressants or mood stabilizers (all *p* > 0.05; Table S6), with a single exception (sdL and mood stabilizer load in the discovery dataset, *p* < 0.05; Table S6a). Similarly, independent t‐tests comparing treated and untreated patients showed no significant differences for most indicators (all *p* > 0.05; Table S7). The few exceptions were: (1) sdL was higher in patients taking antidepressants in the discovery dataset (Table S7a); (2) sdL was lower in patients taking mood stabilizers in the discovery dataset (Table S7a); and (3) SI was higher in patients taking mood stabilizers in the replication dataset (Table S7b). Given the preponderance of non‐significant results, we conclude that medication status had a limited effect on our primary findings. Third, the sample size for the 7T MRI data was limited. In this study, we developed a new analytic framework using data from 272 MDD patients, validated its robustness through cross‐validation, and confirmed its generalizability in an independent sample (*n* = 63). After quality control, 7T MRI data was available for 59 patients within this replication sample. Of these, only 16 were classified into the VMP‐P biotype. This smaller subgroup size reflects the fact that the VMP‐P biotype represents only about a quarter of MDD patients. Nevertheless, future larger‐scale 7T MRI studies are necessary to confirm the utility of abnormal neural activity in the occipital cortex as a key characteristic of the VMP‐P biotype.

Next, a previous study found that the time threshold of small grating stimulus in the VMP test showed no significant difference between recovered depressed patients and healthy subjects [25]. In contrast, our research revealed that acute MDD patients exhibited a significantly elevated time threshold of small‐stimulus (durationS) compared to healthy subjects [27, 28, 29]. Therefore, we suggest that durationS (a static indicator) and the inter‐trial coefficient of variation of the small‐stimulus time threshold (cvS, a dynamic indicator) could serve as baseline predictive indicators and treatment‐monitoring tools. Future studies need to investigate the clinical application of these indicators.

Finally, the VMP paradigm has been applied to the study of multiple neuropsychiatric disorders, including MDD, schizophrenia, and autism [23, 24, 25, 26, 27, 28, 29]. Results indicate that individuals with autism exhibit perceptual hypersensitivity, characterized by significantly shorter time thresholds for both small and large grating stimuli compared to HC subjects [26]. In contrast, patients with MDD and schizophrenia show perceptual impairment, particularly for small stimuli, demonstrated by significantly longer time thresholds for small grating stimuli relative to HCs [23, 24, 27, 28, 29]. Additionally, MDD patients display significantly lower inter‐trial dynamics [29], whereas schizophrenia patients exhibit significantly higher inter‐trial dynamics [23]. These findings suggest the potential future utility of the VMP test for differential diagnosis among these neuropsychiatric disorders.

## 4 Conclusion

This study identifies VMP indices as a promising biomarker for stratifying a novel biotype of depression characterized by impaired visual perception (VMP‐P). Clinically, the VMP‐P biotype was associated with significant cognitive disturbances. The incorporation of rs‐fMRI measurements not only provides a deeper understanding of the neurobiological mechanisms underlying this biotype but also builds a bridge linking impaired neural activity dynamics during visual perception to cognitive dysfunction. These findings have implications for precision rTMS treatment, holding great potential to motivate the discovery of novel therapies that target specific symptom dimensions of MDD. Ultimately, this work contributes to a framework for guiding precision medicine tailored to individual patient profiles.

## 5 Methods

### 5.1 Participants

The discovery dataset consisted of 272 MDD and 303 age‐ and gender‐matched HC subjects (Table 1). The replication dataset consisted of 63 MDD and 79 age‐ and gender‐matched HC subjects (Table S1). The MDD subjects were recruited from the Hangzhou Seventh People's Hospital, from March 2023 to June 2025. The HC subjects were recruited from Zhejiang University and the surrounding communities through advertisements. They were selected to match the MDD group for gender, age, and sociocultural background. The study was approved by the Ethics Committee of Hangzhou Seventh People's Hospital and all research protocols complied with the Declaration of Helsinki. Written informed consent was obtained from each participant after a full description of the study.

All subjects had an education background above the college degree and had normal or corrected to normal vision. Inclusion criteria of the MDD subjects were: i) presence of an acute depressive episode and the diagnosis MDD in accordance with the Diagnostic and Statistical Manual of Mental Disorders, Fifth Edition (DSM‐V) as (a) established by the assessing psychiatrist, and (b) confirmed with Mini International Neuropsychiatric Interview (M.I.N.I.) [70]; ii) clinical symptoms of depression as measured by a Hamilton Depression Rating Scale (HAMD‐17) ≥ 17. Exclusion criteria of MDD subjects were: (i) any other psychiatric disorder, or a mental disorder caused by a physical illness or substance abuse or a personality disorder; (ii) history of traumatic brain injury, epilepsy, or other known organic lesion of the central nervous system; (iii) presence of psychotic symptoms during the depressive episodes; (iv) history of endocrine disease or blood, heart, liver, kidney dysfunction, another medical disorder such as diabetes, or pregnancy; and (v) individuals with claustrophobia or those with metal implants in their bodies, which make them unsuitable for MRI experiments (for the replication dataset). Inclusion criterion of the HC subjects was: (i) no personal or two‐lineage, three generation family history of psychiatric or mental disorder. The exclusion criteria of HC participants were the same as items ii, iii, iv, and v of the exclusion criteria for MDD subjects.

#### 5.1.1 Factors in HAMD‐17 Scale

Factors of HAMD‐17 scale reflected different characteristics in depression. There were five main factors: psychomotor retardation, anxiety/somatization, weight loss, cognitive disorder and sleep disorder. Psychomotor retardation score was calculated by summing the scores of subitems 1, 7, 8, 14 of the HAMD‐17 scale; anxiety/ somatization score was the sum of subitems 10, 11, 12, 15, 17 of the HAMD‐17 scale; weight loss score was the score of subitem 16 of the HAMD‐17 scale; cognitive disorder score was calculated by summing the scores of subitems 2, 3, 9 of the HAMD‐17 scale, and sleep disorder score was the sum of subitems 4, 5, 6 of the HAMD‐17 scale [82, 83].

#### 5.1.2 Measurement of Visual Motion Perception

The visual stimuli were presented on a uniform gray background (luminance: 56 cd/m^2^). As described in our previous publications, the experimental paradigm employed two vertically drifting sinusoidal gratings differing in size (large: 10° diameter; small: 2° diameter) but sharing identical visual parameters (50% contrast, spatial frequency of 1 cycle/°, drift speed of 4°/s. Stimulus presentation was controlled by a Gaussian temporal envelope, with grating duration operationally defined as 1 standard deviation of this envelope. We implemented an adaptive staircase procedure (three‐down/one‐up rule) to dynamically adjust stimulus duration trial‐by‐trial based on participant performance. Threshold measurements were obtained through four interleaved staircases within a 160‐trial block for each stimulus size, ensuring robust psychophysical measurements. This adaptive design resulted in trial‐to‐trial variations in grating movement contingent upon subjects' perceptual judgments. Prior to formal testing, participants completed demonstration and practice trials with auditory feedback provided for incorrect responses to facilitate task understanding.

#### 5.1.3 Participants in Discovery Cohort

Stimulus patterns were generated in MATLAB (MathWorks, Natick, MA) with the Psychophysics Toolbox [84]. The experiment was conducted using a portable computer (1920 ×1080 resolution, 144‐Hz refresh rate, ROG 3) for the visual motion paradigm. Subjects were required to be in a dark environment, sitting at 47 cm away from the LCD screen, with the head stabilized by a chinrest. The edge of the grating was blurred with a Gaussian function [23]. These experiments were conducted in the Hangzhou Seventh People's Hospital.

#### 5.1.4 Participants in Replication Cohort

All stimuli were generated using Psychophysics Toolbox [84] based on MATLAB (MathWorks, Natick, MA, USA) and were shown on display++ monitor (1920 × 1080 resolution, 100‐Hz refresh rate, Cambridge Research System, UK). The viewing distance was 72 cm from the screen, with the head stabilized by a chinrest. The edge of the grating was blurred with a Gaussian function [31]. These experiments were conducted in the psychophysics laboratory of 7T MRI center of Zhejiang university.

#### 5.1.5 Analyses of Static and Dynamic Measures of Psychophysical Performance

The psychophysical experiment recorded the grating duration of each trial, forming a time series. For each participant, we computed the correct rate for different stimulus durations separately for each stimulus size. These values were then fitted to a cumulative Gaussian function, and the static duration threshold corresponding to the 75% correct point on the psychometric function was estimated for each stimulus size (Figure S1a). To quantify the spatial suppression strength, we calculated the spatial suppression index (SI), defined as the difference of log10 thresholds for large vs. small stimuli [24, 25, 26, 27, 28, 29, 30, 31, 35, 36].

(1) $$\mathrm{SI}=\log10\left(\mathrm{large}\;\mathrm{threshold}\right)-\log10\left(\mathrm{small}\;\mathrm{threshold}\right)$$

As traditional static indices used in this paradigm, threshold and SI unable to accurately capture the dynamic changes throughout the task. We used the trial‐by‐trial method to analyze the variability by calculating the absolute value of the difference between the durations of all adjacent small and large stimuli, excluding the consecutively presented maximum and minimum values [23, 29]. The mean and SD indices for this time series were calculated to reflect the variability changes of visual motion perception over time, e.g., the trial‐by‐trial inconsistency of the staircases (Figure S1b). Finally, CV was defined by standard deviation divided by mean value [29]. It reflects the variability of the trials’ duration relative to the overall meaning across all trials.

(2) $$\mathrm{Mean}=\frac{1}{n-1}\Sigma_{i=2}^{n}\left|d\left[i\right]-d\left[i-1\right]\right|$$

(3) $$\mathrm{SD}=\sqrt{\frac{1}{n-1}\Sigma_{i=2}^{n}{\left(\left|d\left[i\right]-d\left[i-1\right]\right|-Mean\right)}^{2}}$$

(4) $$\mathrm{CV}=\frac{SD}{Mean}$$

where *n* is the length of time series *d*.

### 5.2 Block Design Task Measurement

The block design task was administered following standardized procedures outlined in the WAIS‐IV manual [85]. During this assessment, participants were required to reconstruct target figural patterns using bicolored (red and white) blocks within time limits that varied according to item difficulty (30–120 s). Test items were presented in a graded difficulty sequence, with discontinuation rules applied if participants failed to complete two consecutive patterns within the allotted time. Scoring incorporated both accuracy and completion time, with additional bonus points awarded for rapid performance on the final six items. Total scores ranged from 0 to 66 points, with higher scores reflecting superior visuospatial construction ability and perceptual reasoning skills.

### 5.3 Unsupervised Clustering for Visual Perception

#### 5.3.1 Clustering Identification

The assessment of visual motion perception (VMP) in this experiment primarily focused on the following nine metrics: thresholds for small and large stimuli, suppression index (SI), as well as coefficient of variation (CV), standard deviation (SD), and mean values for both stimulus types. Since the visual features exhibited non‐uniform scales and magnitude variations across dimensions, we first applied standardization to normalize the data. We then applied principal component analysis (PCA) to the behavioral task data from MDD patients, identifying two core visual motion perception features that corresponded to principal component 1(PC1) and principal component 2 (PC2) (Figure S2a1,a2).

To determine the optimal number of clusters (k, we employed both the scree plot and silhouette coefficient, evaluating the solution of k values ranging from 1 to 10 (Figure S3). The scree plot identified the point where the sum of squared errors (SSE) began to decrease sharply, while the silhouette coefficient selected the K value corresponding to the highest average score. k ‐means clustering, an effective algorithm suitable for exploring cluster structures in low‐dimensional data, was subsequently applied to the visually‐derived principal components.

#### 5.3.2 Simulation‐Based Significance Testing of the Silhouette Index

To assess the statistical significance of the clustering solutions, we first performed a simulation‐based test. The null hypothesis posited that the observed data originated from a single multivariate normal distribution with no inherent cluster structure. For each candidate cluster number (k = 2–10), we generated 1,000 synthetic datasets and randomly sampled 272/63 participants from a multivariate normal distribution parameterized by the mean and covariance of our actual data. We then applied the same k‐means clustering algorithm to each synthetic dataset and computed the average silhouette index. This procedure yielded a null distribution of silhouette indices for each k. The *p*‐value was defined as the proportion of these simulated indices that exceeded the silhouette index obtained from the empirical data. Cluster solutions with a *p*‐value < 0.05 were considered statistically significant (Figures S4–S6).

#### 5.3.3 Permutation‐Based Significance Testing of the Silhouette Index

As a complementary non‐parametric test, we conducted a permutation‐based analysis. For each k (ranging from 2 to 10), we created 1,000 permuted datasets by randomly shuffling the values of each VMP indices across participants, thereby preserving the marginal distributions while disrupting any multivariate structure. k‐means clustering was performed on each permuted dataset, and the average silhouette index was calculated. The resulting null distribution allowed us to compute a *P* ‐value, defined as the proportion of permuted silhouette indices greater than the observed value. Solutions with *p* < 0.05 were deemed significant (Figures S4–S6).

#### 5.3.4 Assessment of Cluster Stability Using Cross‐Validation

The stability of the cluster assignments was evaluated against minor data perturbations using cross‐validation (Figure S7). We first performed leave‐one‐out cross‐validation, repeating the clustering procedure 272/63 times, each time excluding a single participant. For every run and each k from 2 to 10, the similarity between the new cluster assignments and the original full‐data solution was quantified using the Adjusted Rand Index (ARI). To test robustness against a larger perturbation, we repeated this process using leave‐20%‐out cross‐validation, where 20% of the sample was held out in each iteration.

#### 5.3.5 Correlation‐Based Approach for Cross‐Dataset Clustering Validation

First, a clustering analysis (using the k‐means algorithm) was performed on one dataset, resulting in two final clusters. The mean values of all feature variables within each of these two clusters were calculated. For each sample *x_i_* in the other dataset, the Pearson correlation coefficients between the sample and the nine cluster centroids from the first dataset were computed. These coefficients reflect the linear similarity between the feature pattern of the sample and the characteristic patterns of each cluster centroid.

Based on the correlation comparison, each sample *x_i_* was assigned to the cluster represented by the centroid with which it exhibited the higher correlation [16]. The assignment results were then compared against the original clustering results of the second dataset. The proportion of correctly assigned samples, referred to as the consistency accuracy, was calculated. This metric quantifies the extent to which the cluster membership from the original clustering can be reproduced using the stable cluster centroids from the first dataset as reference anchors.

By leveraging the stable cluster centroids of the larger dataset as reference points, this method effectively evaluates the transferability and robustness of clustering patterns across datasets of different scales. It provides an intuitive and computationally feasible measure for assessing cross‐dataset clustering consistency (Table S2).

### 5.4 Neuroimaging

#### 5.4.1 MRI Data Acquisition

MRI experiments were performed in a 7 T whole body MR system (Siemens Healthcare, Erlangen, Germany) with a Nova Medical 32 channel array head coil. Session included rsfMRI and structural MRI (sMRI). RsfMRI scans were acquired with Echo Planar Imaging (EPI) sequence (1.5 mm isotropic resolution) (transverse orientation, TR/TE = 2000/20.6 ms, 160 volumes, slice number = 110, flip angle = 70°, multi‐band acceleration factor = 5, eyes closed, 6 min and 24 s). sMRI scans were obtained using a MP2RAGE sequence (TR/TI1/TI2 = 5000/901/3200 ms, 8 min and 42 s) with 0.7‐mm isotropic resolution [28, 29, 37].

#### 5.4.2 Rs‐fMRI Data Processing and Analysis

Rs‐fMRI data preprocessing processes for replication sample performed in Statistical Parametric Mapping 12 (SPM 12, http://www.fil.ion.ucl.ac.uk/spm/) using the Data Processing and Analysis for Brain Imaging (DPABI) toolbox [86] including: removal of the volumes in first 10 s, realignment, coregistration of anatomical and functional images for each subject, segmentation of the anatomical images using Diffeomorphic Anatomical Registration through Exponentiated Lie algebra algorithm (DARTEL), linear detrend, nuisance covariates regression (with realignment Friston 24‐parameter, white matter and CSF signal) [87] normalization to the standard Montreal Neurological Institute (MNI) space with a resolution of 1.5 mm3 using DARTEL, spatial smoothing with a 3 mm full width at full‐wide‐half‐maximum (FWHM) Gaussian kernel, and band‐pass filtering with standard frequency band (SFB, 0.01–0.1 Hz). Subjects with head movements greater than 3 mm or 3° were excluded. Since smoothing can blur the relationships between adjacent voxels and reduce the precision of voxel signals, which will negatively affect the results of ReHo, smoothing is not performed in the preprocessing before calculating ReHo.

Structural T1‐weighted images were processed using the CAT12 toolbox employing default preprocessing pipelines. Processing steps included: image interpolation, affine and non‐linear normalization, noise reduction, bias field correction, tissue segmentation (GM, WM, CSF), DARTEL‐based normalization to MNI space, image homogeneity checking, and spatial smoothing with an 8 mm FWHM Gaussian kernel applied to GM maps.

Before GMV and rsfMRI analysis, we defined the regions of interests (ROIs). The bilateral hMT+ complex was anatomically defined as corresponding to the hOc5 area identified through cytoarchitectonic probability maps. [Cytoarchitectonic Analysis of the Human Extrastriate Cortex in the Region of V5/MT+: A Probabilistic, Stereot axic Map of Area hOc5]. Left and right V1 were defined by Brodmann Area 17 (BA17) mask, which was obtained from the Brodmann template. Left and right DLPFC were defined by Brodmann Area 46 (BA46) mask, which was obtained from the Brodmann template. The left and right ACC were represented by Cingulum_Ant_L and Cingulum_Ant_R, which was taken from AAL template.

### 5.5 Statistical Analysis

Statistical analyses were performed using SPSS 27 (IBM, USA). Group comparisons between MDD patients were conducted using two‐tailed independent samples t‐tests. Age and gender were included as covariates and their effects were eliminated. Multiple comparisons across VMP indices were corrected using the false discovery rate (FDR) method, with statistical significance defined at 0.05. Associations between variables were assessed using two‐tailed Pearson's correlation analysis. A significance threshold of *p* < 0.05 was applied for all statistical tests. Mediation analyses were conducted using PROCESS macro (version 4.2) for SPSS. The significance of direct and indirect effects was evaluated through bias‐corrected bootstrapping with 5000 resamples. Mediation effects were considered statistically significant when the 95% confidence intervals (CIs) of the indirect effects excluded zero.

## Author Contributions

S. Liang and X. M. Song were responsible for the study concept and the design of the study. Z. Cai analyzed data and created figures. X. Hu, J. Han, and Z. Tan were in charge of the patients' recruitment and assessment. K, Jia, Y. Cai, Y. Li, and D. Yao provided advice and guided the data analysis for the study. Z. Cai, X. Hu, Y‐Y. Li, and Tao Xu conducted human experiments. Z. Cai, S. Liang, and X. M. Song wrote the manuscript. G. Northoff and Z. Lin made substantial contributions to the manuscript and provided critical comments. All authors evaluated and approved the final version of the manuscript.

## Funding

This work was supported by the ST1 2030‐Major Projects (2022ZD0208500), the Zhejiang Provincial Natural Science Foundation (LTGY24H090012 to SGL; LTGY23C090002 to XMS); the Construction Fund of Key Medical Disciplines of Hangzhou; the Zhejiang Provincial Program for the Cultivation of High‐level 800 Innovative Health talents; the Construction Fund of Key Medical Disciplines of Hangzhou; the Zhejiang Provincial Program for the Cultivation of High‐level Innovative Health talents.

## Conflicts of Interest

The authors declare that they have no conflicts of interest.

## Supporting information




**Supporting File**: advs74882‐sup‐0001‐SuppMat.docx.

## Data Availability

The original data that support the findings of this study are available upon reasonable request.
