# Supplementary material for: Distinct Biotypes of Visual Perception in Major Depressive Disorder
Source: Adv Sci (Weinh). 2026 Mar 18;13(31):e74882. doi: 10.1002/advs.74882 (PMC13587965; doi:10.1002/advs.74882)
Supplement: Supplementary file 1 [file ADVS-13-e74882-s001.docx]

Supporting Information

Distinct biotypes of visual perception in major depressive disorder

Zhuoran Cai^#^, Xi-Wen Hu^#^, Yuyang Li^#^, Zheng Lin, Yongjie Li, Yong-Chun Cai, Tao Xu, Ke Jia, Jin-Fang Han, Zhong-Lin Tan, De-Zhong Yao, Xue Mei Song*, Sugai Liang*, Georg Northoff

**Supporting Information**

[Supplemental Tables	1](#_Toc223978821)

[Table S1. Demographics and patient clinical data in the replication dataset.	1](#_Toc223978822)

[Table S2. Correlation-based validation for cross-dataset clustering.	2](#_Toc223978823)

[Table S3a. Clusters k-means centroids for each principal component (PC) in the discovery dataset.	3](#_Toc223978824)

[Table S3b. Clusters k-means centroids for each principal component (PC) in the replication dataset.	3](#_Toc223978825)

[Table S4. Number of patients from different clustering procedures.	4](#_Toc223978826)

[Table S5. Concomitant medications.	5](#_Toc223978827)

[Table S6a. Correlation analyses between biomarker variables and the load codes for antidepressants and mood stabilizers in the discovery dataset.	6](#_Toc223978828)

[Table S6b. Correlation analyses between biomarker variables and the load codes for antidepressants and mood stabilizers in the replication dataset.	6](#_Toc223978829)

[Table S7a. Independent t-tests comparing treated and untreated patients for antidepressants and mood stabilizers in the discovery dataset.	7](#_Toc223978830)

[Table S7b. Independent t-tests comparing treated and untreated patients for antidepressants and mood stabilizers in the replication dataset.	7](#_Toc223978831)

[Supplementary Figures	8](#_Toc223978832)

[Figure S1. Static and variability indices.	8](#_Toc223978833)

[Figure S2. PCA explained variance and loading matrix.	9](#_Toc223978834)

[Figure S3. Optimal number of clusters.	10](#_Toc223978835)

[Figure S4. Simulation-based and permutation-based significance testing of the silhouette index	11](#_Toc223978836)

[Figure S5. Distribution of silhouette coefficients from simulation-based (green) and permutation-based (red) significance testing in the discovery MDD samples across a range of *k* values from 2 to 10.	12](#_Toc223978837)

[Figure S6. Distribution of silhouette coefficients from simulation-based (green) and permutation-based (red) significance testing in the replication MDD samples across a range of *k* values from 2 to 10.	13](#_Toc223978838)

[Figure S7. Assessment of cluster stability using cross-validation.	14](#_Toc223978839)

[Figure S8. Joint principal component analysis (JPCA) in HC together with the two MDD biotypes.	15](#_Toc223978840)

[Figure S9. K-means clustering identified two subgroups from discover (a) and replication (b) MDD datasets.	16](#_Toc223978841)

[Figure S10. Comparison of HAMD-17 scores between VMP-N and VMP-P biotypes.	17](#_Toc223978842)

[Figure S11. Comparison of block design test (BDT) scores in VMP-N and VMP-P biotypes.	18](#_Toc223978843)

[Figure S12. Comparison of neuroimaging measures in hMT+ (a-d) and V1 (e, f) between the two biotypes.	19](#_Toc223978844)

[Figure S13. Correlation matrix shows correlations between all measured in our VMP indices, neuroimaging indicators and symptoms scores in VMP-P biotype.	20](#_Toc223978845)

[Figure S14. The alternative model did not yield the mediation effects on cognitive disturbance scores.	21](#_Toc223978846)

[Figure S15. No significant correlations between V1 fALFF, ReHo, VMP cvS and clinical scores in the VMP-N biotype.	22](#_Toc223978847)

# Supplemental Tables

## Table S1. Demographics and patient clinical data in the replication dataset.

| Variables | MDD patients  (*n* = 63) | Healthy controls  (*n* = 79) | *P* value |
| --- | --- | --- | --- |
| Gender(M/F) | 24/39 | 43/36 | 0.07 |
| Age, years (SD) | 24.8 (6.0) | 24.0 (3.6) | 0.35 |
| HAMD-17 scores (SD) | 21.53 (3.675) | - | - |
| Cognitive disturbance scores (SD) | 4.579(1.732) | - | - |
| Retardation scores (SD) | 7.211(1.532) | - | - |
| Anxiety scores (SD) | 4.860(1.663) | - | - |
| Sleep disturbance scores (SD) | 2.825(1.571) | - | - |
| Weight loss scores (SD) | 0.2456(0.5438) | - | - |

## Table S2. Correlation-based validation for cross-dataset clustering.

|  | **Replication dataset** | **Discovery dataset** |
| --- | --- | --- |
| Consistency Accuracy | 92.06% | 84.19% |

## Table S3a. Clusters k-means centroids for each principal component (PC) in the discovery dataset.

| **Clusters** | **PC1** | **PC2** |
| --- | --- | --- |
| Cluster 1 | -2.875 | -0.02714 |
| Cluster 2 | 0.9398 | 0.008870 |

## Table S3b. Clusters k-means centroids for each principal component (PC) in the replication dataset.

| **Clusters** | **PC1** | **PC2** |
| --- | --- | --- |
| Cluster 1 | -2.641 | 0.4734 |
| Cluster 2 | 0.9758 | -0.1749 |

## Table S4. Number of patients from different clustering procedures.

| **Method** | **Discovery dataset** | | **Replication dataset** | |
| --- | --- | --- | --- | --- |
|  | VMP-P | VMP-N | VMP-P | VMP-N |
| Without PCA | 69 | 203 | 18 | 45 |
| 2 principal components | 67 | 205 | 17 | 46 |

## Table S5. Concomitant medications.

| **Medication status, *n* (%)** | **Discovery dataset** | | **Replication dataset** | |
| --- | --- | --- | --- | --- |
|  | **VMP-P (*n* = 67)** | **VMP-N (*n* = 205)** | **VMP-P (*n* = 17)** | **VMP-N (*n* = 46)** |
| Off Any Medication | 7 | 41 | 2 | 14 |
|  | (10.4) | (10.0) | (11.8) | (30.4) |
| Off Antidepresant Medication | 13 | 63 | 4 | 15 |
|  | (19.4) | (30.7) | (23.5) | (32.6) |
| On > 1 Antidepresant Medication | 6 | 33 | 2 | 4 |
|  | (9.0) | (16.1) | (11.8) | (8.7) |
| **Antidepresants** | 54 | 142 | 13 | 32 |
|  | (80.6) | (69.3) | (76.5) | (69.6) |
| SSRIs, SNRIs | 44 | 130 | 11 | 29 |
|  | (65.7) | (63.4) | (64.7) | (63.0) |
| others | 12 | 31 | 4 | 5 |
|  | (17.9) | (15.1) | (23.5) | (10.9) |
| Both | 2 | 19 | 2 | 2 |
|  | (3.0) | (9.3) | (11.8) | (4.3) |
| **Mood Stabilizers** | 16 | 55 | 3 | 4 |
|  | (23.9) | (26.8) | (17.6) | (8.7) |
| Lithium | 2 | 14 | 2 | 1 |
|  | (3.0) | (6.8) | (11.8) | (2.2) |
| Anticonvulsants | 14 | 43 | 2 | 3 |
|  | (20.9) | (21.0) | (11.8) | (6.5) |
| Both | 0 | 2 | 1 | 0 |
|  | (0.0) | (1.0) | (5.9) | (0.0) |

Concomitant medication data are presented based on subjects’ reports and available clinical information.

## Table S6a. Correlation analyses between biomarker variables and the load codes for antidepressants and mood stabilizers in the discovery dataset.

| **Pearson correlation coefficient** | **Antidepressants** | **Mood Stabilizers** |
| --- | --- | --- |
| durationS | 0.042 | -0.033 |
| durationL | -0.002 | -0.035 |
| SI | -0.049 | -0.004 |
| cvS | -0.110 | 0.009 |
| cvL | 0.034 | 0.061 |
| sdS | 0.052 | -0.007 |
| sdL | 0.037 | **-0.139*** |
| meanS | 0.085 | -0.032 |
| meanL | -0.005 | -0.108 |

Bold font indicates *P* < 0.05.

## Table S6b. Correlation analyses between biomarker variables and the load codes for antidepressants and mood stabilizers in the replication dataset.

| **Pearson correlation coefficient** | **Antidepressants** | **Mood Stabilizers** |
| --- | --- | --- |
| durationS | 0.051 | -0.076 |
| durationL | 0.021 | 0.117 |
| SI | 0.014 | 0.230 |
| cvS | -0.045 | 0.004 |
| cvL | -0.002 | -0.151 |
| sdS | 0.006 | 0.159 |
| sdL | 0.212 | 0.149 |
| meanS | 0.013 | 0.017 |
| meanL | 0.085 | 0.182 |
| fALFF_LhMT+ | -0.046 | -0.207 |
| fALFF_RhMT+ | -0.062 | -0.171 |
| ReHo_LhMT+ | 0.075 | -0.133 |
| ReHo_RhMT+ | -0.028 | -0.109 |
| GMV_LhMT+ | -0.074 | -0.066 |
| GMV_RhMT+ | -0.070 | -0.209 |
| fALFF_V1 | 0.066 | -0.129 |
| GMV_V1 | -0.031 | -0.090 |
| ReHo_LV1 | 0.077 | -0.094 |
| ReHo_RV1 | 0.072 | -0.055 |

## Table S7a. Independent t-tests comparing treated and untreated patients for antidepressants and mood stabilizers in the discovery dataset.

| **T-value of t-test** | **Antidepressants** | **Mood Stabilizers** |
| --- | --- | --- |
| durationS | 0.540 | -0.920 |
| durationL | -0.567 | -1.073 |
| SI | -1.223 | -0.105 |
| cvS | -0.595 | 1.222 |
| cvL | -0.127 | 1.557 |
| sdS | 1.154 | 0.153 |
| sdL | **2.192*** | **-2.335*** |
| meanS | 1.737 | -0.761 |
| meanL | 0.880 | -1.739 |

Bold font indicates *P* < 0.05.

## Table S7b. Independent t-tests comparing treated and untreated patients for antidepressants and mood stabilizers in the replication dataset.

| **T-value of t-test** | **Antidepressants** | **Mood Stabilizers** |
| --- | --- | --- |
| durationS | 0.474 | -0.752 |
| durationL | 0.233 | 0.653 |
| SI | -0.112 | **2.018*** |
| cvS | -0.541 | 0.670 |
| cvL | -0.455 | -0.805 |
| sdS | 0.119 | 0.914 |
| sdL | 1.309 | 0.621 |
| meanS | 0.024 | -0.230 |
| meanL | 0.580 | 0.828 |
| fALFF_LhMT+ | 0.202 | -1.393 |
| fALFF_RhMT+ | -0.527 | -1.035 |
| ReHo_LhMT+ | 1.048 | -1.171 |
| ReHo_RhMT+ | 0.553 | -0.760 |
| GMV_LhMT+ | -0.053 | -1.233 |
| GMV_RhMT+ | 0.107 | -1.518 |
| fALFF_V1 | 1.700 | -0.618 |
| GMV_V1 | -0.377 | 0.208 |
| ReHo_LV1 | 1.688 | -0.451 |
| ReHo_RV1 | 1.500 | -0.244 |

Bold font indicates *P* < 0.05.

# Supplementary Figures

## Figure S1. Static and variability indices.

a**)** Static index: fitting small and large grating duration thresholds and the calculation formula of suppression index (SI). b) Variability index: A process for calculating variability measurements based on visual perception, including mean, standard deviation (SD) and coefficient of variation (CV) values. The purple and yellow columns represent the two 3-down 1-up staircases in the small stimulus grating. The dashed lines illustrate the temporal dynamics of visual motion perception variability.

## Figure S2. PCA explained variance and loading matrix.

a1 and a2**)** The explained sum of the proportion of variance accounted by each principal component (PC). The first two principal components (PC1: 47.87%, PC2: 17.91%; cumulative variance = 65.78%) were retained for subsequent clustering in the discovery MDD samples (a1). In the replication MDD samples, the first two principal components (PC1: 46.26%, PC2: 19.57%; cumulative variance = 65.83%) were retained for subsequent clustering (a2). b1 and b2) The loading matrix illustrates the contribution (weight) of each original VMP indices to the first two principal components in the discovery (b1) and replication (b2) MDD samples.

## Figure S3. Optimal number of clusters.

a1 and a2) The consensus from a distinct elbow in the scree plot confirmed *k* = 2 as optimal in the discovery (a1) and replication (a2) MDD samples. b1 and b2) a peak in the silhouette metric confirmed *k* = 2 as optimal in the discovery (b1) and replication (b2) MDD samples.

## Figure S4. Simulation-based and permutation-based significance testing of the silhouette index

Permutation-based and simulation-based significance testing of the silhouette index in the discovery (a) and replication (b) MDD samples.

## Figure S5. Distribution of silhouette coefficients from simulation-based (green) and permutation-based (red) significance testing in the discovery MDD samples across a range of *k* values from 2 to 10.

## Figure S6. Distribution of silhouette coefficients from simulation-based (green) and permutation-based (red) significance testing in the replication MDD samples across a range of *k* values from 2 to 10.

## Figure S7. Assessment of cluster stability using cross-validation.

The Adjusted Rand Index (ARI) value of leave-one-out cross-validation and leave-20%-out cross-validation in the discovery (a) and replication (b) MDD samples.

## Figure S8. Joint principal component analysis (JPCA) in HC together with the two MDD biotypes.

Notable overlap between HC group and VMP-N biotype under jointly PCA both in the discover (a) and replication (b) MDD datasets.

## Figure S9. K-means clustering identified two subgroups from discover (a) and replication (b) MDD datasets.

a) Discovery MDD dataset. Two MDD patients were assigned to different subgroups when comparing clustering performed on the original data versus clustering based on the top two principal components (PCs). b) Replication MDD dataset. One MDD patient was assigned to a different subgroup between the two clustering methods. In both panels, green triangles mark the MDD patients whose cluster assignment differed between the PCA-based and non-PCA clustering methods.

## Figure S10. Comparison of HAMD-17 scores between VMP-N and VMP-P biotypes.

Total HAMD-17 scores did not differ significantly between the VMP-N and VMP-P biotypes in the discovery (a) and replication (b) MDD samples.

## Figure S11. Comparison of block design test (BDT) scores in VMP-N and VMP-P biotypes.

a**)** Diagram of the block design test (BDT). b**)** In a discovery cohort subset (*n* = 48), BDT scores were significantly lower for the VMP-N biotype patients (*n* = 10) compared to the VMP-P biotype patients (*n* = 38).

## Figure S12. Comparison of neuroimaging measures in hMT+ (a-d) and V1 (e, f) between the two biotypes.

No significant differences were observed between the two MDD biotypes in the left hMT+ for fALFF(a), ReHo(b), or grey matter volume (GMV) (c). In the right hMT+, the VMP-N biotype showed a trend toward reduced GMV compared to the VMP-P biotype (t=1.80, *P* = 0.08) (d). For the V1, the VMP-N biotype demonstrated lower ReHo in the left hemisphere that approached statistical significance (*P* = 0.06) (e), while no differences were found in the right V1 (f).

## Figure S13. Correlation matrix shows correlations between all measured in our VMP indices, neuroimaging indicators and symptoms scores in VMP-P biotype.

## Figure S14. The alternative model did not yield the mediation effects on cognitive disturbance scores.

## Figure S15. No significant correlations between V1 fALFF, ReHo, VMP cvS and clinical scores in the VMP-N biotype.

While the VMP-P biotype (pink, a-f) showed significant correlations between its V1 fALFF, ReHo, and VMP cvS indices and clinical scores, these correlations were absent in the VMP-N biotype (blue, a-f).
